# Diet Quality and Health Service Utilization for Depression: A Prospective Investigation of Adults in Alberta’s Tomorrow Project

**DOI:** 10.3390/nu12082437

**Published:** 2020-08-13

**Authors:** Shelby Marozoff, Paul J. Veugelers, Julia Dabravolskaj, Dean T. Eurich, Ming Ye, Katerina Maximova

**Affiliations:** School of Public Health, University of Alberta, Edmonton, AB T6G 1C9, Canada; marozoff@ualberta.ca (S.M.); Paul.Veugelers@ualberta.ca (P.J.V.); dabvravol@ualberta.ca (J.D.); deurich@ualberta.ca (D.T.E.); mye@ualberta.ca (M.Y.)

**Keywords:** diet quality, nutrition, mental health, depression, mood disorders, prevention

## Abstract

Depression is a leading cause of disability and economic burden worldwide. Primary prevention strategies are urgently needed. We examined the association of diet quality with depression in a large provincial cohort of adults. A past year food frequency questionnaire was completed by Alberta’s Tomorrow Project (ATP) participants enrolled between 2000–2008 (*n* = 25,016; average age 50.4 years) and used to calculate Healthy Eating Index-Canada (HEI-C) 2015 scores. The number of physician visits for depression 2000–2015 was obtained via linkage with administrative health records. Negative binomial regression models assessed the relationship between HEI-C 2015 scores and physician visits for depression, adjusting for confounders. Every 10-unit increase in HEI-C 2015 scores was associated with 4.7% fewer physician visits for depression (rate ratio (RR): 0.95; 95% Confidence Interval (CI): 0.92–0.98). This relationship persisted when participants with physician visits for mental illness prior to cohort enrollment were excluded. Higher quality diets were associated with a lower number of physician visits for depression. Results highlight diet may be an important prevention strategy for reducing the burden of health service utilization for depression.

## 1. Introduction

Depression is the leading global cause of disability, affecting over 300 million individuals worldwide [1]. In Canada, the lifetime prevalence of depression is over 11% [2]. Mental illnesses, of which depression represents approximately 42.5% of the burden [3], place a substantial strain on health care systems. In 2011, mental illnesses cost the Canadian economy over $22.6 billion dollars in direct costs, a value which is predicted to increase to $105.6 billion over the following 30 years [4]. About one in seven (14%) Canadians access health care for a mental illness annually [5]. Mental illnesses accounted for 25.5% of all acute care hospital days and diagnoses of mental illness in 2009/10 and were associated with hospital stays over 2.5 times as long as those not involving a mental health diagnosis [6]. Given the large personal, societal, and economic burden of depression, primary prevention strategies are urgently needed.

Diet has recently received attention as a promising intervention target for prevention of depression. Poor quality diets are a leading contributor to the burden of chronic disease in Canada [7,8]. Compliance with dietary recommendations is poor among Canadians, and consumption of beneficial food groups, such as vegetables and fruit, as well as milk and alternatives, has decreased in recent years [7]. While there have been two systematic reviews that synthesized evidence from randomized controlled trials (RCTs) on the diet-depression relationship [9,10], RCTs included in both reviews focused on dietary interventions in relation to existing depression symptoms (i.e., secondary prevention). To the best of our knowledge, there are no RCTs that examined diet as a potential target for primary prevention of depression. There are several factors that limit the feasibility of the RCT design when it comes to the diet-depression relationship. Complex exposures, such as diet, affect disease risk through multiple systems and pathways, and these effects aggregate over long periods of time [11], thus requiring sufficient follow-up time [12]. This results in additional costs, as well as non-compliance and high dropout rates among participants. Blinding is also problematic (if at all possible) in RCTs of dietary interventions, and these interventions are often examined in high-risk populations (e.g., obese and overweight) rather than the general population.

Given these challenges, evidence from high-quality observational studies is required to understand if diet is a novel intervention target for primary prevention of depression. Emerging evidence from observational studies indicates that this might be true. For example, several systematic reviews and meta-analyses report the link between adherence to high quality, healthy diets with high intakes of vegetables, fruit, whole grains, and healthy fats and reduced consumption of saturated fat, sugar, and red meat (e.g., whether healthy/prudent, Mediterranean, pro-vegetarian, or Tuscan) and as much as 33% lower risk of incident depression [13,14,15,16,17]. While most of the studies appropriately assessed diet quality (as opposed to individual food constituents) using a variety of tools, the assessment of depression has been limited by the use of symptom severity scales rather than other outcome measures, such as healthcare utilization. For example, available observational studies most commonly included symptom severity scales (e.g., Center for Epidemiologic Studies Depression Scale [18], Beck Depression Inventory-II [19], Composite International Diagnostic Interview Short Form [20]), antidepressant use, or self-report of physician diagnoses, with mixed results that vary by age, sex, and diagnosis [14,15]. While symptom severity scales evaluate the presence of mental illness symptoms regardless of whether formal diagnostic criteria are fulfilled, individuals with symptoms of depression and individuals seeking physician-provided mental health care form two separate yet overlapping groups [21], as not all individuals with symptoms of mental illness will seek physician care. There is value in examining whether the association between diet quality and symptoms of depression extends to individuals seeking health care for depression. Filling this gap can contribute to our understanding of the potential for using dietary approaches to reduce the health service utilization burden.

There is an acknowledged need for large prospective investigations that account for a range of relevant confounders, and focus on population-based samples without a previous history of mental health problems [14]. The aim of the present study was to fill this gap and examine the association between diet quality and health service utilization for depression in a large prospective study of community dwelling adults, accounting for a range of relevant confounders.

## 2. Materials and Methods

From 2000 to 2008, Alberta’s Tomorrow Project (ATP) enrolled 29,876 participants into a population-based prospective cohort study of cancer and chronic disease. Participants were considered eligible if they were ages 35–69 years, with no personal history of cancer other than non-melanoma skin cancer, plans to stay in Alberta for at least one year, and able to complete written questionnaires in English [22]. Participants were recruited using random digit dialing mapped to Regional Health Authority boundaries to facilitate balanced recruitment throughout the province. Eligible participants were mailed baseline questionnaires: the Health and Lifestyle Questionnaire (HLQ), the Canadian Diet History Questionnaire (CDHQ-I), and the Past-Year Total Physical Activity Questionnaire (PYTPAQ), which assessed sociodemographic characteristics, diet, and physical activity, respectively [22]. ATP methods have been previously published [22,23,24]. ATP data were linked to Alberta Health administrative health care databases via Personal Health Numbers and covered the years 2000 to 2015. Over 99% of ATP participants were successfully linked after providing valid Personal Health Numbers and consenting to data linkage [25]. ATP study procedures were approved by the Health Research Ethics Board of Alberta (HREBA)—Cancer Committee (HREBA.CC-17-0461). Ethics approval for the linkage to Alberta Health databases and current analyses was obtained from the University of Alberta Health Research Ethics Board (Pro00058561). All ATP participants provided written consents to participating in ATP and allowing healthcare data linkage and long-term follow-ups at the time of enrolment.

The CDHQ-I, a 124-item past year food frequency questionnaire (FFQ) of foods, beverages, and dietary supplements was based on the Diet History Questionnaire from the U.S. National Cancer Institute [26] and adapted to Canadian food availability, brand names, nutrient composition, and food fortification [23,27]. Responses were analyzed using Diet*Calc (version 1.4.2) software (National Cancer Institute, Bethesda, MD, USA) with a nutrient database adapted for the CDHQ-I to measure each participant’s mean daily intake of energy, nutrients, foods, and supplement use. Diet quality was assessed by the Healthy Eating Index Canada (HEI-C) 2015 (Table S1), based on the American Healthy Eating Index (HEI) 2015 scoring criteria [28] and adapted to age- and sex-specific recommendations from Canada’s Food Guide (CFG) 2007 [29]. The HEI-C 2015 assesses two major components of diet: adequacy (sufficiency of intake of healthy foods and nutrients (e.g., total fruits, whole fruits, total vegetables, greens and beans, whole grains, dairy, total protein foods, seafood and plant proteins, and fatty acids)) and moderation (excess consumption of unhealthy foods and nutrients (e.g., refined grains, sodium, added sugars, and saturated fats)). Moderation components are reverse-scored to reward the restriction of consumption the unhealthy components. The HEI-C 2015 score ranges 0–100, with higher scores indicating better diet quality and greater adherence to dietary recommendations.

We also calculated the Modified Mediterranean Diet Score (MMDS), which has nine components and total index score ranges from zero to nine, with higher scores indicating greater adherence to the Mediterranean diet [30]. Each of the nine components is scored either zero or one based on the sex-specific median, with the exception of alcohol which is based on set consumption values. The MMDS components are constituents of the traditional Mediterranean diet, which is associated with a number of beneficial chronic disease outcomes [31,32].

Health service utilization for depression was estimated using hospital discharge abstracts, physician claims, and prescription medication data between cohort enrollment (2000–2008) and 2015. International Classification of Diseases (ICD), Ninth and 10th Revision codes from administrative health databases of hospital inpatient datasets and the primary, secondary, and tertiary diagnosis fields from physician claims were used to identify depression. Additionally, Anatomical Therapeutic Chemical Classification (ATC) codes for antidepressants or mood stabilizers from Alberta Blue Cross (ABC) pharmacy claims or Pharmaceutical Information Network (PIN) dispenses were used (see Table S2 for ICD-9/10 and ATC codes). The codes for hospital discharge abstracts, physician claims, and prescription medication data were drawn from a validated algorithm from the Manitoba Centre for Health Policy’s Regional Health Authority Indicators Atlas [33,34] to identify health service utilization for depression.

We considered a range of potential confounders. Sociodemographic factors included sex; age; region of residence (urban or rural) determined by postal code; family history of gambling, alcohol, or drug addiction (yes/no); household income (<$30,000, $30,000–59,999, $60,000–99,999, ≥$100,000); highest level of education (high school, some university, post-graduate); marital status (attached or unattached); and employment status (full-time, part-time, other). Lifestyle factors included body weight status (underweight/normal weight, overweight, obese); leisure time moderate-to-vigorous physical activity (MVPA) (rounded quartiles: <70.0 min/week, 70.0–209.9 min/week, 210.0–389.9 min/week, ≥390.0 min/week); smoking status (present, former, never); alcohol intake (g/week); use of supplements (yes/no), specifically vitamin D and other (vitamin A, beta carotene, vitamin E, vitamin C, thiamin, riboflavin, niacin, folic acid, calcium, magnesium, iron, zinc, copper, selenium). The Charlson comorbidity index (0, 1, ≥2 comorbidities) was calculated at baseline, using administrative health records and the ICD-9/10 coding algorithm for administrative healthcare data [35].

### Data Analyses

Given the overdispersion (*p* < 0.05) of physician visits for depression, associations between diet quality and health service utilization for depression were calculated using Negative Binomial Regression Models (NBM). The effect of each of the HEI, moderation, adequacy, and MMDS were evaluated sequentially using four separate models. Rate ratios and 95% confidence intervals were derived from unadjusted, parsimonious, and fully adjusted NBMs. Parsimonious models adjusted for covariates commonly identified in the literature: age, sex, annual household income, educational attainment, physical activity, Body Mass Index (BMI), and energy intake. Fully adjusted models included all covariates from the parsimonious models plus rural/urban residence, marital status, family history of addiction, smoking status, disease comorbidities, vitamin D supplement use, other supplement use, and alcohol intake. Missing values for confounding variables were treated as separate covariate categories in the multivariable NBMs, but their estimates are not presented.

Participants were excluded if they had daily caloric intakes (<500 or >5000 kcal) [36], had not completed the three baseline questionnaires (HLQ, PYTPAQ, and CDHQ-I), or had missing dietary data (>10 missing responses on the CDHQ-I). In order to exclude prevalent cases of depression, we excluded participants with one or more physician visits for mental illness (see Table S3 for ICD-9/10 and ATC codes) during four time periods prior to enrollment in the ATP cohort, including three periods of fixed length (six months, one year and two years prior to cohort enrollment (*n* = 2597, 5391 and 12,657, respectively)) and one period of variable length (from October 2000 to cohort enrollment (*n* = 7681)). These “washout” periods were implemented to increase the chance that depression episodes were incident and are recommended for examining chronic, episodic disorders in administrative health databases [37]. Analyses also included models that excluded physician visits for mental illness in the three months following enrollment to account for the possibility of increased health-consciousness immediately following enrollment in the ATP study. All analyses were conducted using STATA statistical software (Stata Corp LP, College Station, Texas, USA. 2007, Release 14). Statistical significance was set at *p* < 0.05.

## 3. Results

A total of 25,016 participants were available for analysis (Supplementary Materials Figure S1). Excluded participants did not significantly differ from those included in the analytic sample in terms of age, gender, geographic residence, education and presence of comorbidities (data not presented). Baseline characteristics of ATP participants are presented in Table 1. Of 25,016 participants included in analysis, 62.8% were female and 65.7% were overweight or obese. More than one-third (31.1%) of participants had one or more physician visits for depression between enrollment in the ATP cohort and end of follow-up in 2015 (Table 1). Participants’ HEI-C 2015 score was, on average, 61.6 ± 11.0 out of 100 (Table 2), while the MMDS score was, on average, 4.3 ± 1.7 out of 10. Overall, 31.1% of participants saw a physician for depression following enrollment.

Table 3 presents the estimated reduction in the number of physician visits for depression for every 10-unit increase in HEI-C 2015 and component scores. After adjusting for all covariates, every 10-unit increase in HEI-C 2015 score was associated with 4.68% fewer physician visits for depression (rate ratio (RR): 0.95; 95% Confidence Interval (CI): 0.92–0.98). Dietary adequacy was also negatively associated with physician visits, leading to 8.78% fewer visits for depression (RR: 0.91; 95% CI: 0.87–0.96). For each 1-unit increase in MMDS score, there was a 2.48% reduction in the number of physician visits for depression (RR: 0.98; 95% CI: 0.96–0.99) (Table 3). Results capturing the association of diet quality measured with HEI-C 2015 or MMDS and health service utilization for depression remained robust regardless of the covariates adjusted for in parsimonious and fully adjusted models.

A similar reduction in the number of physician visits for depression associated with 10-unit increases in HEI-C 2015 scores was observed when participants were excluded in different periods prior to cohort enrollment. In fully adjusted models, participants had 5.39% (RR: 0.95; 95% CI: 0.91–0.98), 5.38% (RR: 0.95; 95% CI: 0.91–0.98), and 8.34% (RR: 0.92; 95% CI: 0.88–0.96) fewer physician visits when we excluded visits in the six months, one year, or variable-length period from 2000 to cohort enrollment, respectively (Table 4). However, the results were no longer significant after excluding physician visits for mental health in the two years prior to enrollment. Lastly, after excluding physician visits in the three months after cohort enrollment, every 10-unit increase in HEI-C 2015 score and adequacy component score was associated with 4.68% (RR: 0.95; 95% CI: 0.92–0.98) and 8.78% (RR: 0.91; 95% CI: 0.87–0.96) fewer physician visits for depression, respectively (Table 4).

## 4. Discussion

Our study provides evidence that a high-quality diet in adulthood is associated with reduced health service utilization, where every 10-unit increase in diet quality as measured with the HEI-C 2015 was associated with a 4.68% reduction in the number of physician visits for depression. We also observed a similar 2.48% reduction in physician visits for depression for every 1-unit increase in MMDS score, which measures adherence to the Mediterranean diet, high in vegetables, fruit, grains, and legumes, and low in meat and dairy. In addition, our results remained robust when participants with pre-existing physician-diagnosed mental illness were excluded from analyses.

The association between diet quality indices and depression in adults was recently summarized in two systematic reviews. One of the two reviews was limited to 29 prospective studies and found that adherence to high-quality (i.e., healthy/prudent, Mediterranean, pro-vegetarian, or Tuscan) and anti-inflammatory diets was associated with 23% and 19% lower risk of depression, respectively [14]. Another systematic review and meta-analysis of 41 observational studies found that individuals in the highest category of adherence to high-quality and Mediterranean diets had 24% and 33% lower risk of incident depression, respectively, compared to those with lowest adherence [15]. The coefficients from our analyses translate into a difference of up to 17.26% for higher versus lower adherence to the HEI-C 2015 (scores >75 vs. <50) and up to 9.38% for the MMDS (scores >6 vs. <4). These results are of similar magnitude to earlier studies and further corroborate the previously reported associations.

Previous studies often have not accounted for baseline depression status [38,39,40], with findings pointing to complex and bidirectional relations between diet, other lifestyle behaviors, and mental disorders and depression in particular. Given the chronic and episodic nature of depression, our exclusion of participants using four periods prior to cohort enrollment sought to mitigate the issue of reverse causation. It is compelling that our results remained significant with exclusions of six months (*n* = 22,419), one year (*n* = 19,625), and all visits between 2000 and cohort enrollment (*n* = 17,335). Although our results were no longer significant following exclusion of participants with any physician visits for mental illness in the two years prior to cohort enrollment, this is likely due to additionally excluding all participants enrolled in the first two years of ATP. This resulted in a substantial reduction of study sample (12,359/25,016 or approximately half) and a loss of statistical power. As suggested by Molendijk et al. (2018), the correction for depression status at baseline may negate the cumulative effects of lifestyle behaviors in the years before the study. Our work in children demonstrated that adherence to established recommendations for diet, physical activity, sleep, and sedentary behavior at age 10–11 years was associated with 56% fewer physician visits for mental illness in the subsequent four years [41]. This reduction was of similar magnitude with and without correction for mental illness at baseline, i.e., exclusion of all children with a mental illness diagnosis before the baseline. As less than 10% of children in the sample met this exclusion criteria, the similar magnitude of the association with and without correction for baseline mental illness corroborates the findings from this study, and suggests that the lack of association after excluding participants with physician visits in the two years prior to cohort enrollment may indeed be a statistical power issue.

We found that the adequacy component of the HEI-C 2015 remained significant following adjustment for confounders, but the moderation component did not. This finding is interesting and corroborates existing literature. In a recent meta-analysis, the highest category of adherence to healthy diets was associated with less depressive symptoms; however, adherence to unhealthy dietary patterns and food groups was not associated with incident depressive symptoms [14]. Taken together, these findings suggest that a diet sufficient in beneficial foods and nutrients may be more important than a diet restrictive in foods and nutrients recommended to be consumed in moderation. To our knowledge, other studies that have used versions of the HEI to investigate the relationship between diet quality and mental illness have not examined or reported the components of adequacy or moderation.

Existing studies also relied predominantly on cross-sectional design and often did not include several potential confounding variables, such as obesity, energy intake, baseline socioeconomic status (income, parents’ educational level), and medical conditions (e.g., diabetes, food allergies, hypothyroidism) that may be correlated with mood or diet [14,15]. Diet is the product of the interplay among a large number of factors and a lack of consistency in the adjustment for confounders leads to errors in inference and makes comparison across studies difficult [14]. We adjusted for a range of relevant confounders identified in the literature and observed that even after the inclusion of confounders in the models, the results remained robust.

The large sample of the general adult population of Alberta with a diverse range of demographic and behavioral characteristics, followed prospectively for up to 14 years of follow-up and low attrition, the nearly complete linkage with administrative databases, little missing data due to rigorous quality control measures, and the use of multiple measures of diet quality are major strengths. Several limitations warrant consideration. First, although we included a wide range of relevant covariates, the possibility of residual confounding remains. Second, the exposures of interest and confounders, with the exclusion of disease comorbidities, were based on self-report. Nonetheless, the HLQ, CDHQ-I, and PYTPAQ were either composed of existing items used in other studies, adapted from a validated questionnaire, or specifically developed and tested for validity and reliability in this cohort, respectively [22]. Third, since ATP recruited participants when they were already aged 35–69 years, this precludes our ability to observe lifetime incidence of depression. Last, our outcome measure was mental health service utilization, which included participants who had sought out and received physician care for a mental illness. Those who encountered barriers to care or received care outside of the medical system (e.g., psychological counselling through community mental health services) were not included, thus underestimating the burden of depression. Additionally, individuals of higher socioeconomic status may have chosen to seek mental health support through private psychological services, which was also not included. However, if misclassification of outcome did occur, the literature suggests that the result is typically an estimate biased toward the null [42].

## 5. Conclusions

We observed an inverse relationship between diet quality measured with two different dietary indices and health service utilization for depression, independent of socio-economic status (SES), other lifestyle behaviors, and disease comorbidities. Given that the diet quality of many Canadians is of poor quality and either stagnating or declining [7,8], interventions to improve the diets of Canadians at the population level may have important implications for reducing the health care burden for depression in addition to reducing the established risk for chronic disease.

## Figures and Tables

**Table 1:** Baseline characteristics of ATP participants (*n* = 25,016).

		Number of Physician Visits		
	Total (*n* = 25,016)	0 (*n* = 17,227)	1–2 (*n* = 3881)	3+ (*n* = 3908)
	% or Mean (SD)	% or Mean (SD)	% or Mean (SD)	% or Mean (SD)
Age (years)	50.39 (9.17)	50.56 (9.21)	50.49 (9.28)	49.50 (8.87)
Sex				
Male	37.18%	42.70%	28.45%	21.52%
Female	62.82%	57.30%	71.55%	78.48%
BMI (kg/m^2^)				
Underweight/normal weight (≤24.9)	34.03%	34.14%	35.02%	32.55%
Overweight (25.0–29.9)	39.11%	40.29%	37.64%	35.36%
Obese (≥30.0)	26.63%	25.37%	27.11%	31.70%
Location				
Rural	23.64%	24.50%	22.78%	20.70%
Urban	76.36%	75.50%	77.22%	79.30%
Smoking status				
Never	45.12%	47.75%	41.07%	37.54%
Former	37.73%	36.96%	38.52%	40.32%
Current	17.13%	15.27%	20.41%	22.11%
Household income				
<$30,000	12.71%	10.89%	14.33%	19.14%
$30,000–59,999	26.89%	26.09%	28.06%	29.25%
$60,000–99,999	31.87%	32.58%	30.92%	29.68%
≥$100,000	26.29%	28.22%	24.19%	19.83%
Highest level of education				
High school	27.49%	26.97%	29.27%	28.02%
Some university	46.94%	46.18%	47.46%	49.80%
Post-graduate	25.56%	26.85%	23.27%	22.16%
Charlson comorbidity index				
0	83.24%	85.52%	80.06%	76.33%
1	14.31%	12.56%	16.52%	19.83%
2+	2.45%	1.92%	3.43%	3.84%
Energy intake (Kcal/day)	1826.81 (730.92)	1840.59 (732.73)	1797.23 (719.15)	1795.47 (732.82)

ATP, Alberta’s Tomorrow Project; BMI, Body Mass Index; SD, Standard Deviation.

**Table 2:** Average HEI-C 2015 and component scores of ATP participants (*n* = 25,016).

Category/Component	Possible Range	Mean (SD)
Overall HEI-C 2015	0–100	61.56 (11.02)
Adequacy	0–60	34.93 (8.88)
Total vegetables and fruit	0–10	8.02 (2.28)
Whole fruits	0–5	4.04 (1.40)
Greens and beans	0–5	2.53 (1.65)
Whole grains	0–10	3.64 (2.30)
Dairy	0–10	5.86 (3.14)
Total protein foods	0–5	3.43 (1.26)
Seafood and plant proteins	0–5	2.42 (1.44)
Fatty acids	0–10	4.98 (2.78)
Moderation	0–40	26.64 (6.33)
Refined grains	0–10	5.29 (2.33)
Sodium	0–10	7.08 (3.39)
Added sugars	0–10	7.86 (2.37)
Saturated fats	0–10	6.42 (2.90)

ATP, Alberta’s Tomorrow Project; HEI-C 2015, Health Eating Index-Canada 2015; SD, Standard Deviation.

**Table 3:** Associations of increases in HEI-C 2015 and MMDS scores with number of physician visits for depression (*n* = 25,016).

	Unadjusted			Parsimonious ^a^			Fully Adjusted ^b^		
	RR (95% CI)	(1-RR)%	*p*-Value	RR (95% CI)	(1-RR)%	*p*-Value	RR (95% CI)	(1-RR)%	*p*-Value
HEI-C 2015 ^c^	0.96 (0.94, 0.99)	3.68%	0.010	0.95 (0.92, 0.98)	5.12%	0.001	0.95 (0.92, 0.98)	4.68%	0.002
Moderation ^c^	0.94 (0.89, 0.99)	6.03%	0.016	0.93 (0.88, 0.99)	6.55%	0.034	0.96 (0.89, 1.02)	4.48%	0.172
Adequacy ^c^	0.97 (0.94, 1.01)	2.75%	0.122	0.92 (0.88, 0.96)	8.10%	<0.001	0.91 (0.87,0.96)	8.78%	<0.001
MMDS ^d^	0.94 (0.92, 0.96)	5.76%	<0.001	0.97 (0.95, 0.99)	2.75%	0.004	0.98 (0.96, 0.99)	2.48%	0.010

95% CI, 95% Confidence Interval; HEI-C 2015, Health Eating Index-Canada 2015; MMDS, Modified Mediterranean Diet Score; RR, rate ratio; ^a^ Adjusted for sex, age, BMI, leisure time moderate-to-vigorous physical activity (MVPA), household income, educational attainment, caloric intake; ^b^ Adjusted for sex, age, BMI, urban/rural location, family history of addiction, leisure time MVPA, smoking status, household income, educational attainment, marital status, employment status, chronic disease comorbidities, use of vitamin D supplements, use of other supplements, weekly alcohol intake, caloric intake; ^c^ Per 10-unit increase in score; ^d^ Per 1-unit increase in score.

**Table 4:** Associations of increases in HEI-C 2015 and MMDS scores with number of physician visits for depression following exclusions.

	Unadjusted			Parsimonious ^a^			Fully Adjusted ^b^		
	RR (95% CI)	(1-RR)%	*p*-Value	RR (95% CI)	(1-RR)%	*p*-Value	RR (95% CI)	(1-RR)%	*p*-Value
Six-Month Exclusion Prior to Enrollment (n = 22,419)									
HEI-C 2015 ^c^	0.96 (0.93, 1.00)	3.58%	0.025	0.94 (0.91, 0.98)	5.60%	0.001	0.95 (0.91, 0.98)	5.39%	0.002
Moderation ^c^	0.94 (0.89, 1.00)	5.85%	0.033	0.92 (0.86, 0.99)	7.69%	0.026	0.93 (0.86, 1.00)	7.00%	0.056
Adequacy ^c^	0.97 (0.94, 1.01)	2.53%	0.202	0.92 (0.87, 0.96)	8.36%	0.001	0.91 (0.86, 0.96)	8.98%	0.001
MMDS ^d^	0.95 (0.93, 0.97)	5.39%	<0.001	0.97 (0.95, 0.99)	2.61%	0.014	0.98 (0.96, 1.00)	2.31%	0.030
One-Year Exclusion Prior to Enrollment (n = 19,625)									
HEI-C 2015 ^c^	0.96 (0.93, 0.99)	4.07%	0.023	0.94 (0.91, 0.98)	5.85%	0.002	0.95 (0.91, 0.98)	5.38%	0.006
Moderation ^c^	0.92 (0.86, 0.98)	8.35%	0.006	0.91 (0.84, 0.98)	9.21%	0.017	0.92 (0.84, 1.00)	8.50%	0.039
Adequacy ^c^	0.98 (0.94, 1.03)	1.91%	0.395	0.92 (0.87, 0.97)	7.96%	0.004	0.92 (0.86, 0.98)	8.12%	0.006
MMDS ^d^	0.94 (0.92, 0.97)	5.58%	<0.001	0.97 (0.95, 0.99)	2.84%	0.017	0.98 (0.95, 1.00)	2.50%	0.036
Two-Year Exclusion Prior to Enrollment (n = 12,359)									
HEI-C 2015 ^c^	0.99 (0.94, 1.03)	1.50%	0.537	0.96 (0.91, 1.01)	4.39%	0.088	0.98 (0.93, 1.03)	2.14%	0.426
Moderation ^c^	0.94 (0.86, 1.02)	6.12%	0.144	0.89 (0.80, 0.99)	11.03%	0.035	0.94 (0.84, 1.05)	6.05%	0.278
Adequacy ^c^	1.01 (0.95, 1.07)	−0.78%	0.796	0.96 (0.89, 1.04)	4.11%	0.290	0.98 (0.90, 1.07)	1.83%	0.660
MMDS ^d^	0.96 (0.93, 0.99)	3.94%	0.013	0.98 (0.95, 1.00)	2.27%	0.160	0.98 (0.95, 1.01)	2.06%	0.203
Variable-Length Exclusion Prior to Enrollment (n = 17,335)									
HEI-C 2015 ^c^	0.92 (0.89, 0.96)	7.61%	<0.001	0.91 (0.87, 0.95)	9.17%	<0.001	0.92 (0.88, 0.96)	8.34%	<0.001
Moderation ^c^	0.88 (0.82, 0.94)	11.87%	<0.001	0.87 (0.79, 0.95)	13.45%	0.002	0.87 (0.79, 0.96)	12.85%	0.005
Adequacy ^c^	0.94 (0.90, 0.99)	5.74%	0.022	0.87 (0.81, 0.93)	13.04%	<0.001	0.87 (0.81, 0.93)	12.76%	<0.001
MMDS ^d^	0.94 (0.92, 0.97)	5.91%	<0.001	0.97 (0.95, 1.00)	2.75%	0.043	0.97 (0.95, 1.00)	2.57%	0.059
Exclusion of Visits Three Months Following Enrollment (n = 25,016)									
HEI-C 2015 ^c^	0.96 (0.94, 0.99)	3.75%	0.009	0.95 (0.92, 0.98)	5.08%	0.001	0.95 (0.92, 0.98)	4.68%	0.002
Moderation ^c^	0.94 (0.89, 0.99)	6.11%	0.015	0.94 (0.88, 1.00)	6.41%	0.039	0.96 (0.89, 1.02)	4.48%	0.172
Adequacy ^c^	0.97 (0.94, 1.01)	2.81%	0.115	0.92 (0.88, 0.96)	8.06%	<0.001	0.91 (0.87, 0.96)	8.78%	<0.001
MMDS ^d^	0.94 (0.92, 0.96)	5.82%	<0.001	0.97 (0.95, 0.99)	2.75%	0.004	0.98 (0.96, 0.99)	2.46%	0.011

95% CI, 95% Confidence Interval; HEI-C 2015, Health Eating Index-Canada 2015; MMDS, Modified Mediterranean Diet Score; RR, rate ratio; ^a^ Adjusted for sex, age, BMI, leisure time MVPA, household income, educational attainment, caloric intake; ^b^ Adjusted for sex, age, BMI, urban/rural location, family history of addiction, leisure time MVPA, smoking status, household income, educational attainment, marital status, employment status, chronic disease comorbidities, use of vitamin D supplements, use of other supplements, weekly alcohol intake, caloric intake; ^c^ Per 10-unit increase in score; ^d^ Per 1-unit increase in score.

## Supplementary Materials

The following are available online at https://www.mdpi.com/2072-6643/12/8/2437/s1: Figure S1: Flow chart for the inclusion and exclusion of Alberta’s Tomorrow Project (ATP) cohort (2000–2008); Table S1: Scoring Criteria for the Healthy Eating Index-Canada 2015; Table S2: ICD 9/10 and ATC Codes Identifying Physician Visits for Depression; Table S3: ICD 9/10 and ATC Codes Identifying Physician Visits for Mental Illness.
