# Supplementary material for: Diet Quality and Health Service Utilization for Depression: A Prospective Investigation of Adults in Alberta’s Tomorrow Project
Source: Nutrients. 2020 Aug 13;12(8):2437. doi: 10.3390/nu12082437 (PMC7468802; doi:10.3390/nu12082437)
Supplement: Supplementary file 1 [file nutrients-12-02437-s001.pdf]

Supplemental Figure S1

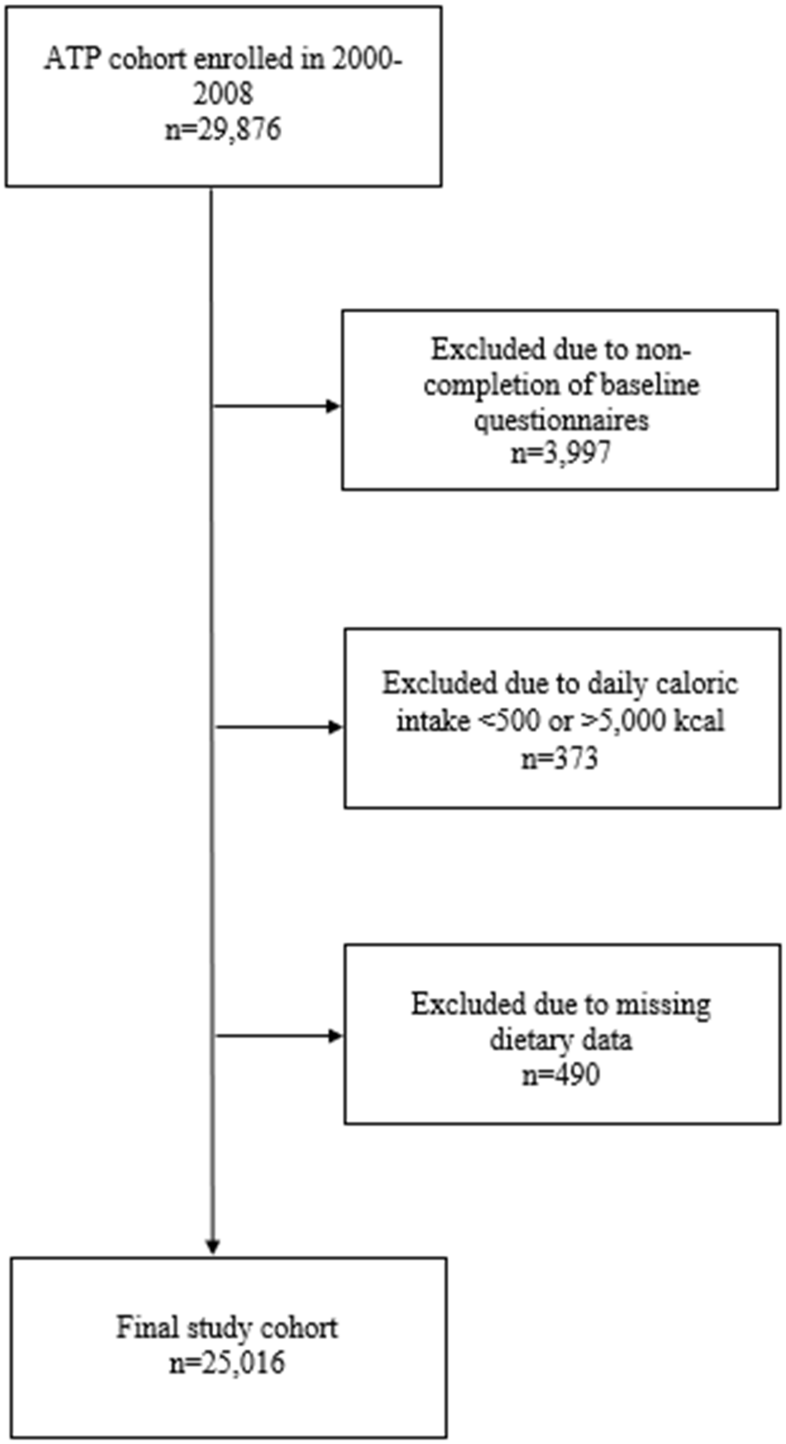

Figure S1. Flow chart for the inclusion and exclusion of Alberta's Tomorrow Project (ATP) cohort (2000-2008)

Table S1. Scoring Criteria for the Healthy Eating Index-Canada 2015

Maximum Points	Component	Standard for Maximum Points	Standard for Minimum Score of Zero
Adequacy			
10	Total Fruits and Vegetables	7-8 servings	No Fruits and Vegetables
5	Whole Fruits	1.47-1.68 servings	No Whole Fruits
5	Greens and Beans	0.74-0.84 servings	No Dark Green Vegetables or Legumes
10	Whole Grains	3-4 servings	No Whole Grains
10	Dairy	2-3 servings	No Dairy
5	Total Protein Foods	2-3 servings	No Protein Foods
5	Seafood and Plant Proteins	0.64-0.96 servings	No Seafood or Plant Proteins
10	Fatty Acids	(PUFA+MUFA)/SFA≥2.5	(PUFA+MUFA)/SFA≤1.2
Moderation			
10	Refined Grains	<50% of grains refined	100% of grains refined
10	Sodium	≤UL	≥2x UL
10	Added Sugars	≤6.5% of energy	≥26% of energy
10	Saturated Fats	≤8% of energy	≥16% of energy

Abbreviations: HEI-C 2015, Healthy Eating Index Canada 2015; MUFA, Monounsaturated Fatty Acids; PUFA, Polyunsaturated
Fatty Acids; SFA, Saturated Fatty Acids; UL, Tolerable Upper Intake Level

Table S2. ICD 9/10 and ATC Codes Identifying Physician Visits for Depression

ICD-9/10 and ATC codes
Inpatient data
ICD-9: 296.2-296.8; 300.4; 309; 311;
ICD-10: F31; F32; F33; F34.1; F38.0; F38.1; F41.2; F43.1; F43.2; F43.8; F53.0; F93.0
**OR**
Claim data
ICD-9: 296; 309; 311
**OR**
Inpatient data
ICD-9: 300;
ICD-10: F32.0; F34.1; F40; F41; F42; F44; F45.0; F45.1; F45.2; F48; F68.0; F99
**AND**
ABC/PIN dispense data within 6 months of hospitalization
ATC: N03AB02; N03AB52; N03AF01; N05AN01; N06A
**OR**
Claim data
ICD-9: 300
**AND**
ABC/PIN dispense data within 6 months of claims
ATC: N03AB02; N03AB52; N03AF01; N05AN01; N06A

Abbreviations: ABC, Alberta Blue Cross; ATC, Anatomical Therapeutic Chemical Classification; ICD, International Classification of Diseases; PIN, Pharmaceutical Information Network

Table S3. ICD 9/10 and ATC Codes Identifying Physician Visits for Mental Illness[a]

**ICD-9/10 and ATC codes**
Inpatient data<br>ICD-9: 296.2-296.8; 300.4; 309; 311;<br>ICD-10: F31; F32; F33; F34.1; F38.0; F38.1; F41.2; F43.1; F43.2; F43.8; F53.0; F93.0<br>**OR**<br>Claim data<br>ICD-9: 291; 292; 293; 294; 295; 296; 297; 298; 300 excluding 300.14, 300.15, 300.16, 300.19, 300.8, 300.81, 300.82; 301.1; 303; 304; 305.2-305.9; 309; 311<br>**OR**<br>Inpatient data<br>ICD-9: 300;<br>ICD-10: F32.0; F34.1; F40; F41; F42; F44; F45.0; F45.1; F45.2; F48; F68.0; F99<br>**AND**<br>ABC/PIN dispense data within 6 months of hospitalization<br>ATC: N03AB02; N03AB52; N03AF01; N05AN01; N06A<br>**OR**<br>Claim data<br>ICD-9: 300<br>**AND**<br>ABC/PIN dispense data within 6 months of claims<br>ATC: N03AB02; N03AB52; N03AF01; N05AN01; N06A

Abbreviations: ABC, Alberta Blue Cross; ATC, Anatomical Therapeutic Chemical Classification; ICD, International Classification of Diseases; PIN, Pharmaceutical Information Network

[a] Includes visits for depression, anxiety disorders, affective disorders, organic and non-organic psychoses, and substance use disorders.
